# The Prognostic Utility of Cytokines in Hospitalized COVID-19 Patients

**DOI:** 10.2478/jccm-2023-0025

**Published:** 2023-11-14

**Authors:** Ákos Vince Andrejkovits, Adina Huțanu, Ervin József Susányi, Valentina Negrea, Anca Meda Văsieșiu

**Affiliations:** Doctoral School of Medicine and Pharmacy, I.O.S.U.D., George Emil Palade University of Medicine, Pharmacy, Science, and Technology of Targu Mures, Romania; Department of Laboratory Medicine, George Emil Palade University of Medicine, Pharmacy, Science, and Technology of Targu Mures, Romania; Center for Advanced Medical and Pharmaceutical Research, Targu Mures, Romania; First Infectious Disease Clinic of Targu Mureș, Mureș County Clinical Hospital, Romania; Department of Infectious Disease, George Emil Palade University of Medicine, Pharmacy, Science, and Technology of Targu Mures, Romania

**Keywords:** cytokines, prognosis, COVID-19

## Abstract

**Introduction:**

The severity of COVID-19 relies on several factors, but the overproduction of pro-inflammatory cytokines remains a central mechanism. The aim of this study was to investigate the predictive utility of interleukin (IL)-6, IL-8, IL-10, IL-12, tumor necrosis factor alpha (TNF-α), and interferon gamma (IFN-γ) measurement in patients with COVID-19.

**Material and Methods:**

We prospectively enrolled 181 adult patients with COVID-19 admitted to the 1^st^ Infectious Disease County Hospital Târgu Mureș from December 2020 to September 2021. Serum cytokine levels were measured and correlated with disease severity, need for oxygen therapy, intensive care unit (ICU) transfer, and outcome.

**Results:**

We found significantly higher serum levels of IL-6, IL-8, and IL-10 in patients with severe COVID-19 and in those with a fatal outcome. The logistic regression analysis showed a significant predictive value for IL-8 regarding disease severity, and for IL6 and IL-10 regarding ICU transfer and fatal outcome.

**Conclusions:**

Serum levels of IL-6, IL-8, and IL-10 were significantly increased in patients with COVID-19, but their predictive value regarding disease severity and the need for oxygen therapy was poor. We found IL-6 and IL-10 to have a good predictive performance regarding ICU transfer and fatal outcome.

## Introduction

COVID-19 has quickly spread across the globe, affecting over 600 million people with more than 6.4 million deaths worldwide [1, 2]. Most SARS-CoV-2-infected patients develop only a mild, self-limiting disease. However, approximately 15–20% of COVID-19 patients develop severe pneumonia, and 5–10% need intensive care treatment [3,4,5].

Severe COVID-19 induces a broad range of immunological events, leading to the overproduction of pro-inflammatory cytokines and the alteration of normal antiviral immune responses [6]. This pathological state triggered by the SARS-CoV-2 virus, termed cytokine release syndrome (CRS), is characterized by the rapid and prolonged elevation in the serum levels of more than 20 inflammatory cytokines and chemokines [7]. CRS often induces acute respiratory distress syndrome (ARDS) and secondary hemophagocytic lymphohistiocytosis, which can lead to extensive tissue damage, multi-organ failure, and death [8,9]. Pro-inflammatory cytokines and hyperinflammation are believed to be key factors in this abnormal systemic immune response, which can be associated with significant morbidity and even death [9]. CRS may also be caused by a complex cellular immune dysregulation that is associated with lymphopenia, decreased numbers of circulating T, B, and NK cells, and drastic changes in myeloid cell lines [10,11,12]. Identifying the biomarkers produced during this state of hyperinflammation could be very helpful for understanding and early identification of this phenomenon, for outcome prediction and appropriate management [7,13].

Studies have shown that serum levels of interleukin (IL)-1b, IL-1RA, IL-2, IL-6, IL-7, IL-8, IL-9, IL-10, IL-17, IL-18, tumor necrosis factor alpha (TNF-α), and interferon gamma (IFN-γ) are significantly increased in COVID-19 [9,14,15,16], and some of them (IL-6, IL-8, and TNF-α) are independently correlated with disease severity [4].

IL-6 is a critical cytokine in COVID-19 associated with CRS [8]. This glycoprotein, secreted by macrophages, is a pro-inflammatory cytokine with an important role in the regulation of homeostasis [8,17]. CRS seems to be associated with IL-6 dysregulation, elevated IL-6 levels being associated with respiratory failure and poor prognosis [18,19]. Another study showed that IL-6 and C-reactive protein levels on the first day of admission were predictors of mortality in severe COVID-19 [20].

Another pro-inflammatory cytokine is IL-8, a potent chemotactic factor that attracts neutrophils, basophils, and T-cells during the inflammatory process. IL-8 is released from several cell types in response to inflammation [21,22,23], and it is believed to serve as a biomarker to indicate the prognosis of COVID-19 [22]. It was also demonstrated that in severe COVID-19 with ARDS, IL-8 may contribute to the formation of neutrophil extracellular traps, which were found in postmortem pulmonary specimens of patients with COVID-19 [23,24].

IL-10 is traditionally classified as an anti-inflammatory and immunosuppressive cytokine, produced by various myeloid- and lymphoid-derived immune cells. Studies suggest that it may also have prognostic utility regarding COVID-19 outcomes [1,25]. Other studies revealed that elevated serum IL-10 levels in patients with COVID-19 can be both an anti-inflammatory mechanism and an immunosuppressive biomarker [26,27]. In COVID-19, a higher level of IL-10 was found to be associated with a more severe disease [28].

A study that analyzed cytokine levels in patients with COVID-19 found that the serum levels of IL-6, IL-8, and IL-10 were significantly higher in patients with a more severe form of the disease. Furthermore, a score combining the levels of these three cytokines was highly predictive of progression to severe COVID-19 [29].

The aim of this study was to investigate the relationship between the serum levels of a panel of pro-inflammatory cytokines consisting of IL-6, IL-8, IL-10, IL-12, TNF-α, IFN-γ and disease severity, need for oxygen therapy, ICU transfer and fatal outcome in patients with confirmed COVID-19.

## Materials and methods

### Study design

We performed a single-center prospective cohort study including 181 adult patients admitted to the 1st Infectious Diseases County Hospital of Târgu Mureș, Romania between December 2020 and September 2021. All patients were diagnosed with COVID-19, confirmed by positive real-time polymerase chain reaction (RTPCR). Patients under the age of 18 years, pregnant women, patients with HIV infection or AIDS, different types of cancer, or connective tissue disorders, transplant recipients and patients with incomplete or missing data were excluded from the study.

For each patient, clinical and demographical parameters (sex and age) were obtained from the medical records and introduced into a database. We collected data on comorbidities, serum cytokine levels, clinical severity of COVID-19, need for oxygen therapy, length of stay, ICU transfer and outcome. Cytokine (IL-6, IL-8, IL-10, IL-12, TNF-α and IFN-γ) serum levels were measured in the first 1–3 days of admission. The blood samples were collected in the first 24 h after hospitalization and, where appropriate, in the first 24 h after reclassification into another severity group, at any time during hospitalization. In the statistical analysis we used the blood samples collected in the first 24 hours after admission.

Patients were split into two age groups: **≤**60 years old and older 60.

We compared cytokine serum levels between cases with COVID-19 of different severities to evaluate their predictive value of in relation to the disease severity, need for oxygen therapy, ICU transfer and outcome. We also compared serum cytokine levels according to sex, age, comorbidities and length of stay.

### COVID-19 severity classification

The severity of COVID-19 was defined based on the World Health Organization’s guidance on the clinical management of COVID-19 published on November 23, 2021. Accordingly, ‘mild stage’ was defined as a disease with few symptoms (low fever, cough, fatigue, anorexia, shortness of breath, and myalgias), without evidence of viral pneumonia or hypoxia. ‘Moderate stage’ was defined as a disease with fever and respiratory symptoms, associated with pulmonary imaging findings but no signs of severe pneumonia and an oxygen saturation (SpO_2_) of ≥94% on room air. ‘Severe stage’ was defined as the presence of severe pneumonia, plus one of the following: respiratory rate of >30 breaths/min, severe respiratory distress, or SpO_2_ <94% on room air. Based on this classification, study cases were divided into two categories: 1) non-severe (mild and moderate stage); 2) severe (severe stage).

### Sample collection and cytokine measurement

The blood samples were collected by venous puncture into vacuum collectors with clot accelerator, were centrifuged and the serum was cryopreserved until analysis at −20 °C. The samples were transported to the Laboratory of Humoral Immunology of the Center for Advanced Medical and Pharmaceutical Research, where serum cytokine levels were measured using xMAP technology with a customized Human Magnetic beads panel for cytokine detection (EMD Millipore Corporation) on a Flexmap 3D analyzer. The laboratory procedures were performed following the manufacturers’ recommendations. For all cytokines, the measuring intervals ranged between 0.64 and 10,000 pg/ml, and the intra-assay coefficient of variation was below 3.5%.

### Statistical analysis

We performed a descriptive analysis, expressing categorical variables with numbers and percentages; numerical variables with mean, median, range and inter-quartile range (IQR). The distribution of the data was assessed using the Kolmogorov-Smirnov test and analyzed using the non-parametric Mann-Whitney U test. Each parameter was estimated using a 95% confidence interval (95% CI). Multivariate logistic regression analysis was performed for disease severity, ICU transfer and outcome. The predictive ability of the studied parameters regarding ICU transfer and outcome was estimated using the receiver operating characteristic curve (ROC) and the area under the curve (AUC), where an AUC 0.5 indicated no predictive ability, a value of 0.8 was considered good, and a value of 1.0 was considered perfect. All statistical analyses were performed using IBM SPSS Statistics for Windows version 26 (Armonk, NY: IBM Corp). A *p* value <0.05 was considered statistically significant.

### Ethics

The study was approved by the ethics committees of the “George Emil Palade” University of Medicine, Pharmacy, Science and Technology of Târgu Mureș (1237/08.01.2021) and of the Mureș County Clinical Hospital (19038/21.12.2020). Written informed consent was obtained from each patient before inclusion, and full anonymity was preserved for all participants.

## Results

### Baseline characteristics

A total of 181 hospitalized patients were enrolled in the study. At admission, 87 (48.06%) patients had severe COVID-19, and 48 (26.51%) were reclassified from moderate to severe COVID-19 during hospital stay. The clinical and demographic characteristics of the patients are presented in Table 1.

**Table 1.** Clinical and demographic characteristics of the cohort, grouped by disease severity

**Parameter**	**Non-severe (n = 94)**	**Severe (n = 87)**	**p value**
Female sex	44	35	0.229
Male sex	50	52	
Age (median)	64 (21–86)	64 (24–90)	0.575
Length of stay (days)	12 (1–32)	14 (4–36)	<0.001
Hypoxemia requiring oxygen therapy	49 (52.12%)	85 (97.7%)	**<0.001**

**Comorbidities**			
Arterial hypertension	56 (59.57%)	54 (62%)	0.424
Cardiovascular diseases	22 (23.4%)	18 (20.68%)	0.398
Insulin-dependent diabetes	6 (6.38%)	15 (17.24%)	0.230
Non-insulin-dependent diabetes	14 (14.89%)	11 (12.64%)	0.413
COPD	3 (3.19%)	2 (2.29%)	0.537
Asthma	4 (4.25%)	4 (4.59%)	0.596
Hyperlipidemia	30 (31.91%)	25 (28.73%)	0.381
Obesity	25 (26.59%)	38 (43.67%)	**0.012**
Chronic renal disease	3 (3.19%)	8 (9.19%)	0.083
ICU transfer	8 (8.51%)	27 (31.03%)	**<0.001**

**Outcome**			
Discharged	87 (92.55%)	70 (80.45%)	**0.014**
Died	7 (7.44%)	17 (19.54%)	

COPD= chronic obstructive pulmonary disease; ICU=intensive care unit.

### Age and sex distribution of the patients

The mean age of the patients was 64 years (range 21–90). There were 57 severe and 57 non-severe cases among patients above 60 years; 30 severe and 37 non-severe cases among patients below 60 years (*p* = 0.3). We found no correlation between age and disease severity (r = 0.042; *p* = 0.229). In total, 114 out of 181 (62.98%) patients were above 60 years, and we found significantly higher serum levels of IL-6 (*p* = 0.015), IL-8 (*p* = 0.06), IL-10 (*p* = 0.006), and TNF-α (*p* <0.0001) in these patients.

We found a significant difference in the distribution of sexes between patients above and below 60 years, with a male predominance among younger patients (*p* = 0.003). We found no correlation between sex and serum cytokine levels.

### Ethnicity

Population distribution by ethnicity: 177 (97,7%) white patients and 4 (2,2%) Indo-Aryan (gipsy). We found no correlation between ethnicity and disease severity, outcome or ICU transfer requirement.

### Comorbidities

The patients’ comorbidities included arterial hypertension, diabetes mellitus, chronic obstructive pulmonary disease (COPD), asthma, pulmonary fibrosis, hyperlipidemia, obesity and chronic renal and liver disease. IL-6 (p = 0.035), IL-8 (p <0.0001), IL-10 (p = 0.009), and TNF-α (*p* <0.0001) serum levels were significantly higher in patients with arterial hypertension. We found no statistically significant differences when comparing serum cytokine levels according to the presence of obesity, COPD, asthma and chronic renal disease.

### Length of stay

The average length of hospital stay was 13 days, with an equal distribution between sexes. Hospital stay was significantly longer in patients over 60 years (*p* = 0.016) (mean 13.5 days, range 5–36) than in patients below 60 years (mean 11.9 days, range 1–32 days). The length of stay was not significantly associated with outcome (*p* = 0.244). A longer hospital stay was also found in patients who required ICU transfer (*p* <0.0001), and the length of stay was significantly influenced by the presence of respiratory failure (*p* <0.0001). Furthermore, a positive correlation was found between the length of stay and serum levels of IL-10 (r = 0.273, *p* = 0.0002), IL-6 (r = 0.217, *p* = 0.003) and IL-8 (r = 0.187, *p* = 0.011).

### Disease severity

With the exception of IL-12 (*p* = 0.341) and IFN-γ (*p* = 0.926), the serum levels of all other cytokines showed significant differences between cases with severe (*n* = 87) and non-severe (*n* = 94) COVID-19. We found significantly higher levels of IL-6 (*p* = 0.002), IL-8 (*p* = 0.002), IL-10 (*p* = 0.006) and TNF-α (*p* = 0.039) in severe COVID-19.

### Need for oxygen therapy

In total, 134 out of 181 (74.03%) patients needed oxygen therapy, 90 (67.16%) of which were over 60 years. We found a positive correlation between the need for oxygen therapy and age (r =0.163; *p* = 0.038). Patients who needed oxygen therapy presented significantly higher serum levels of IL-6 (*p* = 0.016), IL-8 (*p* = 0.001), IL-10 (*p* <0.0001) and TNF-α (*p* = 0.002). Furthermore, we found a negative correlation between serum levels of IL-6 (r = −0.278; *p* = 0.0002), IL-10 (r = −0.262; *p* = 0.0004), TNF-α (r = −0.175; *p* = 0.018) and SpO_2_ measured on admission.

### ICU transfer

In total, 35 (19.33%) patients required ICU transfer, 25 (71.42%) from the >60 age group and 10 (28.58%) from the **≤**60 age group. The average length of ICU stay was 9 days (range 1–24 days). IL-6, IL-8 and IL-10 serum levels were significantly higher in patients who required ICU transfer (Table 2).

**Table 2.** Serum cytokine levels in patients with COVID-19 according to the need for ICU transfer

	**ICU transfer (n = 35)**	**No ICU transfer (n = 146)**	**p value**
IFN-γ (pg/ml)	0.78 (0.64–2.98)	0.64 (0.64–1.84)	0.403
IL-6 (pg/ml)	29.08 (4.69–189.1)	0.64 (0.64–7.7)	<0.0001
IL-8 (pg/ml)	11.99 (2.41–37.81)	1.15 (0.64–5.54)	<0.0001
IL-10 (pg/ml)	30.45 (13.16–70.25)	6.29 (1.16–23.78)	<0.0001
IL-12 (pg/ml)	0.64 (0.55–1.48)	0.64 (0.61–1.21)	0.591
TNF-α (pg/ml)	21.72 (15.47–33.69)	19.73 (11.78–30.77)	0.147

ICU=intensive care unit, IFN-γ=interferon gamma, IL=interleukin, TNF=tumor necrosis factor

### Outcome

Of the 114 patients over 60 years, 94 (82.46%) were discharged and 20 (17.54%) have died, while of the 67 patients below 60 years, 63 (94.03%) were discharged and 4 (5.97%) have died (*p* = 0.02). A total of 24 (13.25%) patients died during hospital stay, of which 13 (54.16%) were female and 11 (45.84%) were male. With the exception of IFN-γ and IL-12, all the other studied cytokines showed significantly higher serum levels in non-survivors. We observed a similar trend for IL-12, but the results did not reach the significance level (Table 3).

**Table 3.** Serum cytokine levels in survivors vs. non-survivors

	**Survivor (n = 157)**	**Non-survivor (n = 24)**	**p value**
IFN-γ (pg/ml)	0.64 (0.64–1.87)	0.94 (0.57–2.97)	0.662
IL-6 (pg/ml)	0.64 (0.64–7.85)	38.56 (15.24–714.3)	<0.0001
IL-8 (pg/ml)	1.1 (0.64–5.41)	18.89 (7.49–54.89)	<0.0001
IL-10 (pg/ml)	6.76 (0.64–23.82)	42.45 (27.19–80.85)	<0.0001
IL-12 (pg/ml)	0.64 (0.59–1)	0.93 (0.57–2.54)	0.054
TNF-α (pg/ml)	18.98 (12.29–29.47)	25.8 (18.65–35.97)	0.013

### Predictive utility of cytokines

We performed a multivariate regression analysis, in which the dependent variables were represented by severity (non-severe vs. severe), survival (deceased vs. alive), and ICU transfer (transferred to the ICU vs. not transferred to the ICU) and the independent variables were represented by the different interleukins. The aim of the multivariate regression analysis was to assess the prognostic utility of interleukins based on the dependent variables. An estimated risk value of >1, expressed through the odds ratio (OR), signifies a risk or unfavorable prognosis.

The results of the multivariate regression analysis showed that depending on the ‘severity’ variable, IL-8 had a prognostic value for disease severity, increased levels being associated with severe forms (Table 4).

**Table 4.** Results of the logistic regression analysis regarding the disease severity

**Variable**	**Disease severity**		
	**Odds Ratio**	**95% CI**	**p value**
IFN-γ	0.9505	0.8830 to 1.0232	0.1769
IL-10	1.0070	0.9980 to 1.0161	0.1297
IL-12	1.0395	0.9661 to 1.1184	0.2995
IL-6	0.9994	0.9974 to 1.0015	0.5799
IL-8	1.0364	1.0008 to 1.0743	0.0414
TNF-α	1.0072	0.9797 to 1.0355	0.6103

Depending on the ‘survival’ variable, IL-8, IL-6 and IL-10 had prognostic value, increased levels being associated with death. Of note, these three interleukins had higher values in deceased patients even at the bivariate analysis (Table 5).

**Table 5.** Results of the logistic regression analysis regarding the outcome

**Variable**	**Outcome**		
	**Odds Ratio**	**95% CI**	**p value**
IFN-γ	0.8594	0.7494 to 0.9856	0.0302
IL-10	1.0122	1.0026 to 1.0219	0.0126
IL-12	1.0513	0.9849 to 1.1223	0.1330
IL-6	1.0046	1.0008 to 1.0093	0.0399
IL-8	1.0430	1.0175 to 1.0693	0.0009
TNF-α	1.0054	0.9626 to 1.0501	0.8085

Depending on the ‘ICU transfer’ variable, IL-8, IL-6 and IL-10 had prognostic value, increased levels being observed in those transferred to the ICU. Similarly, to the ‘survival’ variable, these three interleukins had higher levels in those transferred to the ICU even at the bivariate analysis (Table 6).

**Table 6.** Results of the logistic regression analysis regarding ICU transfer

**Variable**	**ICU transfer**		
	**Odds Ratio**	**95% CI**	**p value**
IFN-γ	0.9212	0.8257 to 1.0278	0.1419
IL-10	1.0114	1.0018 to 1.0210	0.0196
IL-12	0.9977	0.9287 to 1.0719	0.9503
IL-6	1.0080	1.0006 to 1.0164	0.0409
IL-8	1.0353	1.0121 to 1.0590	0.0026
TNF-α	0.9871	0.9508 to 1.0248	0.4959

**Fig. 1.**
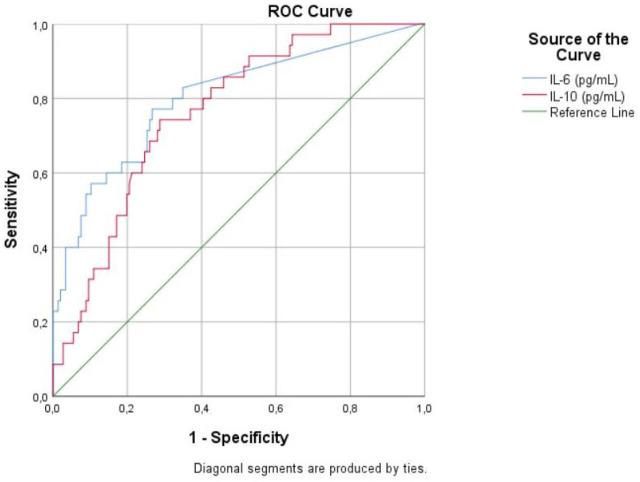
Receiver Operating Characteristic curve (ROC) analysis for IL-6 and IL-10 regarding the ICU transfer

ROC curve analysis for IL-8 regarding disease severity yielded an AUC of 0.632 (95% CI 0.550–0.714; *p* = 0.002), with a cut-off level of 5.36 pg/ml. The analysis showed a good predictive ability for IL-6 (AUC 0.801; 95% CI 0.713–0.889; *p* <0.001) and IL-10 (AUC 0.763; 95% CI 0.684–0.842; *p* <0.001) regarding ICU transfer (Figure 1).

**Fig. 2.**
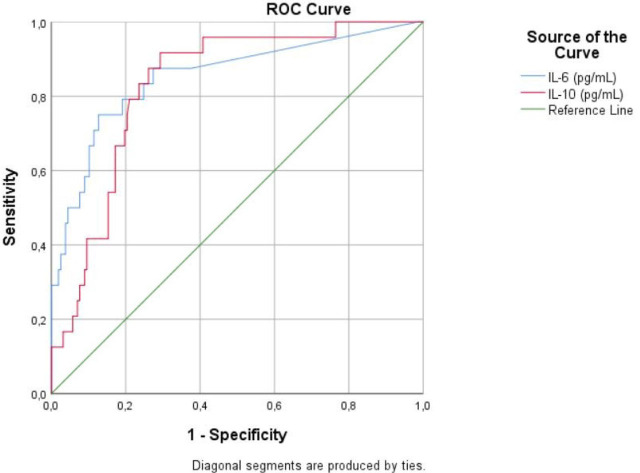
Receiver Operating Characteristic curve (ROC) analysis for IL-6 and IL-10 regarding the outcome prediction

ROC curve analysis also showed a good predictive ability for IL-6 (AUC 0.853; 95% CI 0.759–0.946; *p* <0.001) and IL-10 (AUC 0.831; 95% CI 0.756–0.906; *p* <0.001) regarding outcome (Figure 2).

Cut-off levels and AUC values for IL-6 and IL-10 regarding patient outcome are shown in Table 7.

**Table 7.** Cut-off values and ROC analysis for biomarkers with statistical significance in predicting outcome in COVID-19 patients

**Parameter**	**Cut-off value**	**AUC (95% CI)**	**p value**	**Sensitivity (%)**	**Specificity (%)**
IL-6	20.14pg/ml	0.853 (0.793–0.901)	<0.0001	75.00	87.26
IL-10	18.00pg/ml	0.831 (0.768–0.882)	<0.0001	91.67	70.70

## Discussion

Outcome prediction for the appropriate management of patients with COVID-19 is a serious challenge. The severity of COVID-19 depends on several factors, but the pathogenesis of CRS and hyperinflammation remains in center of the interest. In these settings, a bio-marker with high sensitivity and specificity can guide the clinician to identify high-risk patients for close follow-up and appropriate management.

Hormonal differences between men and women may cause differences in ACE-2 expression and the production of cytokines, and may also affect mortality [30,31]. In women, estrogen has a protective effect on the immune system [31]. In a retrospective study conducted on 548 inpatients with COVID-19, Qin *et al*. found higher levels of IL-10 and TNF-α in male patients [32]. Although there were more men in our study group, we found no connection between the patients’ sex and serum cytokine levels.

Studies have shown that age may be the most important risk factor for severe COVID-19 and its complications [31,33]. Furthermore, older patients can have an increased production of pro-inflammatory cytokines, which can lead to CRS [34]. Although in our study IL-6, IL-8, IL-10 and TNF-α serum levels were significantly higher in subjects >60 years, we found no correlation between age and disease severity.

Comorbidities, such as hypertension and diabetes, can also affect the production of pro-inflammatory cytokines including IL-2R, IL-10 and TNF-α [32]. Our findings are in agreement with these reports, as serum levels of IL-6, IL-8, IL-10, TNFα were significantly elevated in patients with arterial hypertension.

Studies suggest that obesity-induced inflammation can modify innate and adaptive immune responses, resulting in a greater vulnerability to infection [35,36]. In our study, we found a significant association between obesity and disease severity (*p* = 0.012).

Hypoxemia, longer hospital stays and ICU transfer were significantly associated with severe COVID-19. However, there were no significant differences between patients with severe and non-severe disease regarding hypertension and diabetes mellitus.

A longer hospital stay was associated with the presence of respiratory failure and ICU transfer, and serum levels of IL-6, IL-8, and IL-10 were higher in these patients.

Guo *et al*. identified 11 cytokines with a predictive role in severe COVID-19 including granulocyte colony-stimulating factor (G-CSF), hepatocyte growth factor (HGF), IL-6, IL-7, IL-8, IL-10, IL-18, interferon gamma-induced protein 10 (IP-10), macrophage colony-stimulating factor (M-CSF), monokine induced by interferon-gamma (MIG) and stem cell growth factor-beta (SCGF-β) [37]. Of these, IL-6, IL-8 and IL-10 were found to have with poor predictive value regarding disease severity [38].

In our study, elevated IL-6, IL-8, and IL-10 serum levels were correlated with the presence of respiratory failure. An injured lung can be a major source of IL-6 production, which may explain correlations observed between cytokine serum levels and the need for oxygen therapy [4,39]. TNF-α is linked to bronchial hyperresponsiveness and is involved in the deterioration of respiratory epithelium by cytokines [8]. We found TNF-α serum levels to be correlated with the presence of hypoxemia. However, our results suggest that cytokines have a poor predictive value regarding the need for oxygen therapy.

Studies suggest there is a positive correlation between ICU transfer and elevated serum levels of IL-6, IL-8 and TNF-α. Also, higher TNF-α levels are associated with a longer ICU stay in men [13,18]. We found significantly increased serum levels of IL-6, IL-8, IL-10 and TNF-α in patients who required ICU transfer.

In previous studies, increased TNF-α, IL-1Ra, IL-6, IL-8, IL-15 and IL-10 serum levels were associated with higher mortality in COVID-19 [14]. Furthermore, IL-1, IL-6 and TNF-α produced by macrophages have been reported as pathogenic factors in the excessive inflammatory response in COVID-19. Research suggests that the inflammatory cascade and increased secretion of pro-inflammatory cytokines in the lower airways may be responsible for tissue destruction in various organs [18,40,41]. In this study, we found elevated IL-6, IL-8, IL-10 and TNF-α serum levels in patients with fatal outcome and who required oxygen therapy. Of the studied cytokines, IL-6 had the best predictive ability for outcome (AUC 0.853; 95% CI 0.759–0.946; *p* <0.001).

IL-6 is reported to have a unique role in the cytokine storm related to COVID-19. Increased IL-6 serum levels can modulate the activity of natural killer cells and were found to be associated with other immune dysregulations. Previous studies have explored its predictive value regarding disease severity, ICU transfer and outcome [1,3,42]. In this study, IL-6 had a good predictive ability for ICU transfer (AUC = 0.801) and outcome (AUC = 0.853).

IL-8 is involved in the activation and recruitment of neutrophils in COVID-19, and studies suggest that it may be a biomarker of ARDS [14,43]. In our study, IL-8 demonstrated a poor predictive ability for outcome, ICU transfer or the need for oxygen therapy. These findings suggest that compared to the other cytokines we studied, IL-8 is a poor predictor of COVID-19 severity.

IL-10, an anti-inflammatory cytokine, amplifies viral sepsis in severe COVID-19, probably through overactivation and proliferation [14,44]. Some studies have found that IL-10 can be used to predict poor outcome in COVID-19 [1,13]. Our study confirms these findings, as IL-10 showed a good predictive capacity for outcome.

Similarly, to our study, Zhou *et al*. reported an optimal IL-6 cut-off value of 26.09 pg/ml for the prediction of outcome [45]. In another study, the cut-off value was established at 101.64 pg/ml [20]. We found a cutoff value of 20.14 pg/ml with a sensitivity of 75.0% and a specificity of 87.2%, which may be explained by the different cohort type, comorbidities and distribution of severe cases.

## Conclusions

Our findings indicate that IL-6, IL-8, and IL-10 serum levels are significantly increased in COVID-19 but are associated with poor predictive ability regarding disease severity and the need for oxygen therapy. From the studied cytokines, IL-6 and IL-10 seem to be independent predictors for ICU transfer and outcome, with IL-6 having a better predictive performance. A limitation of the study is the small number of patients and a rather limited level of originality when addressing the cytokine role in the COVID-19 severity. We believe though that by diversifying the endpoints it provides a useful addition to the literature on this subject.
